# Foraging plasticity in seabirds: A non-invasive study of the diet of greater crested terns breeding in the Benguela region

**DOI:** 10.1371/journal.pone.0190444

**Published:** 2018-01-31

**Authors:** Davide Gaglio, Timothée R. Cook, Alistair McInnes, Richard B. Sherley, Peter G. Ryan

**Affiliations:** 1 FitzPatrick Institute of African Ornithology, DST-NRF Centre of Excellence, University of Cape Town, Rondebosch, South Africa; 2 Institute of Ecology and Environmental Sciences, Evolutionary Ecophysiology Team, University Pierre and Marie Curie, Bâtiment Paris, France; 3 DST/NRF Centre of Excellence at the FitzPatrick Institute of African Ornithology, Department of Zoology, Nelson Mandela University, Summerstrand, South Africa; 4 Environment and Sustainability Institute, University of Exeter, Penryn Campus, Penryn, Cornwall, United Kingdom; University of Reunion Island, RÉUNION

## Abstract

Marine predators, such as seabirds, are useful indicators of marine ecosystem functioning. In particular, seabird diet may reflect variability in food-web composition due to natural or human-induced environmental change. Diet monitoring programmes, which sample diet non-invasively, are valuable aids to conservation and management decision-making. We investigated the diet of an increasing population of greater crested terns *Thalasseus bergii* in the Western Cape, South Africa, during three successive breeding seasons (2013 to 2015), when populations of other seabirds feeding on small pelagic schooling fish in the region were decreasing. Breeding greater crested terns carry prey in their bills, so we used an intensive photo-sampling method to record their diet with little disturbance. We identified 24,607 prey items from at least 47 different families, with 34 new prey species recorded. Fish dominated the diet, constituting 94% of prey by number, followed by cephalopods (3%), crustaceans (2%) and insects (1%). The terns mainly targeted surface-schooling Clupeiformes, with anchovy *Engraulis encrasicolus* the most abundant prey in all three breeding seasons (65% overall). Prey composition differed significantly between breeding stages and years, with anchovy most abundant at the start of the breeding season, becoming less frequent as the season progressed. The proportion of anchovy in the diet also was influenced by environmental factors; anchovy occurred more frequently with increasing wind speeds and was scarce on foggy days, presumably because terns rely in part on social facilitation to locate anchovy schools. The application of this intensive and non-invasive photo-sampling method revealed an important degree of foraging plasticity for this seabird within a context of locally reduced food availability, suggesting that, unlike species that specialise on a few high-quality prey, opportunistic seabirds may be better able to cope with reductions in the abundance of their preferred prey.

## Introduction

Upper trophic-level predators can be used as indicators of marine food supplies as changes in their life history traits, such as their diet, often reflect variations in the environment (e.g., [1]). Large, high-quality diet data-sets provide a basis to examine short- and long-term environmental changes, providing a better understanding of ecosystem functioning, and giving the potential to support ecosystem-based management [2, 3]. In highly dynamic and often unpredictable marine ecosystems such as upwelling systems, top predators like seabirds must adjust their diet and foraging ecology in response to seasonal and inter-annual changes in food availability. Thus, seabird diet may reflect variability in food-web composition due to natural or human-induced environmental change [4, 5].

Although seabirds have evolved several life-history characteristics to help buffer scarce and/or unpredictable forage resources, they may still be affected negatively by reductions in food availability (e.g., [6]). Such negative impacts are particularly evident during breeding, when prey availability and localized environmental factors affect seabirds because of range restrictions imposed by central-place foraging [7]. Variation in food supplies around breeding colonies can have strong impacts on species that carry only one prey at a time (single-prey loaders) with short foraging ranges, such as terns [8, 9]. Some specialist species target a few high-quality prey species [10], making them vulnerable to stochastic availability of their preferred prey and other abiotic constraints [9, 11]. Other species are more versatile, enabling them to buffer changes resulting from climate change and/or human exploitation in the availability of their favoured prey species by switching to other, more readily available, but often lower quality food [12–14]. Such opportunistic feeding can be sufficient to maintain adult condition and survival, but is not optimal for the growth and survival of chicks [15, 16].

The greater crested tern *Thalasseus bergii* is a coastal seabird with an extensive breeding range that includes the Benguela Upwelling Region. It is an inshore forager that mainly acquires food by diving to < 1 m below the sea surface [17]. When breeding, adults predominantly return to their nests with single prey items carried in their bills [18]. Research on Australian populations suggests that higher quality prey is more important for chick provisioning than for adult maintenance [19]. In southern Africa, little is known about the greater crested tern’s foraging ecology; both studies of the species’ diet (based on chick regurgitations and direct observations of chick provisioning) found a predominance of schooling Clupeiformes fish [20, 21]. These pelagic fish species, mostly anchovy *Engraulis encrasicolus* and sardine *Sardinops sagax*, are a major food source for three seabird species endemic to the Benguela region, African penguins *Spheniscus demersus*, Cape cormorants *Phalacrocorax capensis* and Cape gannets *Morus capensis*. Both sardine and anchovy are also targeted by a large, industrial purse-seine fishery [22]. Populations of these three endemic seabird species have decreased since the 1980s, largely due to reductions in pelagic fish availability [6, 23], resulting in all three being listed as globally threatened with extinction [24]. Competition with fisheries and environmental changes have caused a spatial mismatch between pelagic fish distributions and seabird breeding colonies [23, 25–29]. In contrast to recent population trajectories of these Benguela endemics, the greater crested tern population has increased over the last two decades [23, 30], a period for which there is no published information on this species’ diet. We report the species' prey composition over three breeding seasons (2013–2015) to assess how the foraging ecology of greater crested terns in the Benguela region has been influenced by recent habitat conditions associated with regional shifts in pelagic fish availability. By using a novel photo-sampling technique we were able to collect large sample sizes in a non-invasive manner [31], which allowed us to assess how greater crested tern diet varied with breeding stage, season and localized environmental factors.

## Methods

Research was conducted on Robben Island (33°48’S, 18°22’E), off the south-west coast of South Africa, where ca 90% of the South African population of greater crested tern breeds [23, 32]. During this study (2013–2015), two colonies of different sizes were established in 2013 and 2014, whereas in 2015 only one large colony was established. The two colonies were located in the same areas each year in 2013–2014, with the ‘single-species’ colony comprising about 7,500 and 8,000 pairs in 2013 and 2014, respectively. The other colony included Hartlaub’s gulls *Chroicocephalus hartlaubii* (hereafter termed the ‘mixed-species’ colony) and was situated within the human settlement on the island; it contained ca 2,500 pairs of terns in 2013, 800 in 2014 and 8,200 in 2015, when the entire island population bred in this area.

Prey carried by terns visiting the breeding colony was recorded during three successive breeding seasons: February–May 2013, January–May 2014 and March–May 2015. Prey identity was assessed with a non-invasive photo-sampling technique, which consists of taking a sequence of photos (termed a ‘photo-set’) of adult birds flying towards the colony with prey, allowing for accurate estimation of anchovy standard length (SL) (methods are described in detail in [31]). Photo-sampling was carried out for several days per week (range 1–7 days) from incubation until the chicks fledged. Generally, each photo-sampling session lasted from 1 to 3 hr and was carried out randomly throughout the day between civil dawn (ca 6–7h00) and dusk (ca 18–20h00) from February–May, a period when light levels make photography possible and when terns breed [33] (S1 Table). An index of breeding stage (pre-laying, incubation, and different stages of chick-rearing) was obtained from a combination of visual inspection at the two colonies and detailed observations of images from camera-traps placed within the colony (camera-traps were set to photograph nest contents every day for 1h in the morning and 30 min in the afternoon) (S1 Fig). The breeding phenology of greater crested terns is highly synchronous [17], and for each colony the week of median hatching was estimated. Breeding stage was classified as incubation (during which time prey returned are used for displaying or courtship purposes), early-provisioning (the mean week when chicks are provisioned in the nest cup), mid-provisioning (the mean week subsequent to early provisioning when chicks begin to leave the nest) and late-provisioning (the period when adults provision mobile chicks, invariably in crèches).

### Environmental factors

Several environmental factors were investigated to assess their influence on prey returned by terns to their breeding colonies. Visibility was recorded as the presence or absence of fog within one hour preceding the photo-sampling and was determined by the observer's ability to distinguish landmarks at a known distance (500 m). Hourly wind speed measurements were obtained from Robben Island weather station (South African Weather Service). Tidal stage, defined as hours before and after high tide (range from -5 to 6), was obtained from www.tides.mobilegeographics.com.

### Data analysis and statistics

Prey were classified into pelagic species (inhabiting the pelagic zone, including pelagic-neritic and pelagic-oceanic species, as well as demersal species with pelagic juvenile stages), demersal fish (inhabiting the sea floor) and bentho-pelagic fish (species living throughout the water column). Where possible, prey were identified to species or family; very small translucent fish were classified as fish larvae (mainly anchovy and beaked sandfish *Gonorynchus gonorynchus* larvae) and their number recorded. The proportion of each prey species in the total number of prey species photographed was calculated, with an annual sample size of at least 3,000 prey items being considered optimal [31].

Chi-squared goodness-of-fit tests were used to assess differences in diet composition between incubation and the chick-rearing period within and between seasons. Because anchovy constituted most of the terns’ diet (65%), ANOVAs followed by Tukey post-hoc tests were performed to compare mean anchovy SL between seasons, breeding stages and colonies. Anchovy SL was log_10_ transformed to fulfil assumptions of normality because the Shapiro test for normality (function *shapiro*.*test*; R package ‘nortest’) indicated that the untransformed data were not normally distributed. In order to visualize the importance of each prey group in relation to breeding stage, differences in species composition were plotted in an ordination via non-metric multidimensional scaling (NMDS) using the R package ‘vegan’ [34]. NMDS is an unconstrained ordination technique that allows the use of dissimilarity measures appropriate for ecological data sets, with the use of distance measures to graphically represent relationships between groups with similar diets in multidimensional space [35, 36]. The Bray–Curtis dissimilarity measure, after the Hellinger transformation [37], was used with NMDS ordination (with a maximum of 100 iterations) to compare weekly diet variation according to breeding stage, among the sampling weeks for the two colonies and all years combined. Interpretation of the NMDS plot relies on relative distances between objects, with objects closer together being more similar. The function *ordiellipse* was used to plot ellipses in order to show the centroid corresponding to each breeding stage [35, 38].

The influence of environmental variables on the probability of anchovy capture (dominant prey) by greater crested terns was assessed using generalised additive models (GAM) to accommodate potential non-linear relationships between continuous explanatory variables and the response [39]. We included the presence or absence of anchovy for each individual observation as a binary response (i.e., 1 = anchovy, 0 = other prey) and used a binomial error distribution with a logit link function. Smoother terms included two environmental variables (wind velocity and tide) and a temporal variable (week), calculated as the chronological week number commencing from the last week of January (i.e., the beginning of the breeding season; [17]). Week number was included to control for seasonal variation in anchovy abundance, which may be influenced by the movement of recruits through their foraging area during the tern’s breeding season [40, 41]. Explanatory variables expressed as factors included visibility (clear vs foggy days), breeding stage, colony and year. GAMs were fitted using the R package 'mgcv' [39] with smoother functions generated using penalised regression splines, an upper limit of 6 on the effective degrees of freedom (edf) to prevent overfitting, and the degree of smoothness determined by the generalized cross validation criteria [39]. Collinearity between continuous explanatory variables was assessed using variance inflation factors [42] with a minimum threshold set to 3. The significance level was set at P < 0.05 for all statistical tests. All computations were carried out in R [43].

## Results

### Diet of tern chicks and incubating adults

Photo-sampling of tern diet was carried out on 125 days, capturing ca 50,000 photo-sets that yielded images of 24,607 prey items. Annual sampling sizes were 2,954 prey in 2013, 9,738 in 2014 and 11,915 in 2015. Prey brought back to the colony were dominated by fish (96%), with < 4% comprised of invertebrates: three cephalopod species, three crustacean species and four insect taxa (Table 1 and Table 2; S2 Fig). Among the fish prey, at least 38 families and 53 genera, including 13 pelagic, 34 demersal and 6 bentho-pelagic fish species were recorded (Table 1 and Table 2). Pelagic fish accounted for 94% of all prey (Table 1 and Table 2). Anchovy was the dominant prey followed by juveniles of redeye round-herring *Etrumeus whiteheadi*, Atlantic saury *Scomberesox saurus*, long-snout pipefish *Syngnathus temminckii*, sardine and horse mackerel *Trachurus capensis* (Table 1). Fish larvae accounted for 0.5% of the diet (Table 2; S2 Fig).

**Table 1:** Numbers and proportions of prey species (and families) photographed in the bills of adult greater crested terns returning to colonies on Robben Island between 2013 and 2015.

Common name	Scientific name	Family	Habitat	N 2013	%	N 2014	%	N 2015	%	N TOT	TOT %
Anchovy	*Engraulis encrasicolus*	Engraulidae	Pelagic	2420	81.92%	6527	67.03%	7259	60.93%	16206	65.518%
Redeye round-herring	*Etrumeus whiteheadi*	Dussumieriidae	Pelagic	92	3.11%	648	6.65%	1817	15.25%	2557	10.338%
Atlantic saury	*Scomberesox saurus*	Scomberesocidae	Pelagic	45	1.52%	1245	12.78%	368	3.09%	1658	6.703%
Long-snout pipefish	*Syngnathus temminckii*	Syngnathidae	Bentho-pelagic	41	1.39%	597	6.13%	227	1.91%	865	3.497%
Sardine	*Sardinops sagax*	Clupeidae	Pelagic	57	1.93%	69	0.71%	419	3.52%	545	2.203%
Horse mackerel	*Trachurus capensis*	Carangidae	Pelagic	57	1.93%	150	1.54%	201	1.69%	408	1.649%
Beaked sandfish	*Gonorynchus gonorynchus*	Gonorynchidae	Demersal	35	1.18%	23	0.24%	49	0.41%	224	0.906%
Beaked sandfish larvae	*Gonorynchus gonorynchus*	Gonorynchidae	Pelagic	1	0.03%	13	0.13%	103	0.86%	127	0.513%
Cape silverside	*Atherina breviceps*	Atherinidae	Pelagic	3	0.10%	69	0.71%	126	1.06%	198	0.800%
Southern mullet	*Liza richardsonii*	Mugilidae	Demersal	7	0.24%	21	0.22%	89	0.75%	117	0.473%
Cape hake	*Merluccius capensis*	Merlucciidae	Demersal	14	0.47%	27	0.28%	35	0.29%	76	0.307%
Elf	*Pomatomus saltatrix*	Pomatomidae	Pelagic	6	0.20%	23	0.24%	38	0.32%	67	0.271%
Sole spp.	*Austroglossus* spp. (2)	Soleidae	Demersal	37	1.25%	21	0.22%	5	0.04%	63	0.255%
Gaper	*Champsodon capensis*	Champsodontidae	Demersal	6	0.20%	13	0.13%	39	0.33%	58	0.234%
Klipfish spp.	Clinidae spp. (6)	Clinidae	Demersal	11	0.37%	14	0.14%	33	0.28%	58	0.234%
Agile klipfish	*Clinus agilis*	Clinidae	Demersal	1	0.03%	0	0.00%	0	0.00%	1	0.004%
Bull klipfish	*Clinus taurus*	Clinidae	Demersal	1	0.03%	2	0.02%	0	0.00%	3	0.012%
Bearded klipfish	*Pavoclinus mentalis*	Clinidae	Demersal	10	0.34%	1	0.01%	2	0.02%	13	0.053%
Grass klipfish	*Pavoclinus graminis*	Clinidae	Demersal	0	0.00%	0	0.00%	1	0.01%0.00%	3	0.012%
*Cancelloxus* klipfish	*Cancelloxus* sp.	Clinidae	Demersal	4	0.14%	0	0.00%	1	0.01%	5	0.020%
Snaky klipfish	*Blennophis anguillaris*	Clinidae	Demersal	0	0.00%	3	0.03%	4	0.03%	7	0.028%
Soldierfish sp.		Holocentridae	Demersal	0	0.00%	2	0.02%	45	0.38%	47	0.190%
Bluebottle fish	*Nomeus gronovii*	Nomeidae	Pelagic	3	0.10%	33	0.34%	9	0.08%	45	0.182%
Cape gurnard	*Chelidonichthys capensis*	Triglidae	Demersal	13	0.44%	5	0.05%	25	0.21%	43	0.174%
Blenny sp.		Blenniidae	Demersal	2	0.07%	2	0.02%	34	0.29%	38	0.154%
Lantern fish	*Lampanyctodes hectorus*	Myctophidae	Pelagic	1	0.03%	1	0.01%	21	0.18%	23	0.093%
Goby spp.	*Sufflogobius* spp. (2)	Gobiidae	Demersal	7	0.24%	0	0.00%	2	0.02%	9	0.036%
Bearded goby	*Sufflogobius bibabartus*	Gobiidae	Demersal	9	0.30%	0	0.00%	14	0.12%	23	0.093%
Chub mackerel	*Scomber japonicus*	Scombridae	Pelagic	0	0.00%	4	0.04%	18	0.15%	22	0.089%
Shyshark spp.	*Haploblepharus *spp. (2)	Scyliorhinidae	Demersal	3	0.10%	2	0.02%	11	0.09%	16	0.065%
Grenadier sp.		Macrouridae	Demersal	0	0.00%	0	0.00%	12	0.10%	12	0.049%
Southern conger	*Gnathophis capensis*	Congridae	Demersal	3	0.10%	2	0.02%	5	0.04%	11	0.044%
Southern conger larvae	*Gnathophis capensis*	Congridae	Pelagic	0	0.00%	0	0.00%	1	0.01%	1	0.004%
Dolphinfish	*Coryphaena hippurus*	Coryphaenidae	Pelagic	0	0.00%	10	0.10%	0	0.00%	10	0.040%
Rockfish sp.	*Sebastes* sp.	Sebastidae	Demersal	1	0.03%	1	0.01%	4	0.03%	6	0.024%
Rocksucker	*Chorisochismus dentex*	Gobiesocidae	Demersal	1	0.03%	2	0.02%	3	0.03%	6	0.024%

**Table 2:** Numbers and proportions of prey species (and families) photographed in the bills of adult greater crested terns returning to colonies on Robben Island between 2013 and 2015.

Common name	Scientific name	Family	Habitat	N 2013	%	N 2014	%	N 2015	%	N TOT	TOT %
Silver scabbardfish	*Lepidotus caudatus*	Trichiuridae	Bentho-pelagic	1	0.03%	1	0.01%	3	0.03%	5	0.020%
Largehead hairtail	*Trichiurus lepturus*	Trichiuridae	Bentho-pelagic	0	0.00%	1	0.01%	3	0.03%	4	0.016%
Pufferfish sp.		Tetraodontidae	Demersal	0	0.00%	2	0.02%	3	0.03%	5	0.020%
Redfingers	*Cheilodactylus fasciatus*	Cheilodactylidae	Demersal	2	0.07%	2	0.02%	0	0.00%	4	0.016%
Snake eel sp.		Ophichthidae	Demersal	0	0.00%	0	0.00%	3	0.03%	4	0.016%
Snake eel sp. larvae		Ophichthidae	Pelagic	0	0.00%	1	0.01%	0	0.00%	1	0.004%
Codlet sp.		Bregmacerotidae	Pelagic	1	0.03%	0	0.00%	3	0.03%	4	0.016%
Kingklip	*Genypterus capensis*	Ophidiidae	Demersal	1	0.03%	1	0.01%	1	0.01%	3	0.012%
Carpenter seabream	*Argyrozona argyrozona*	Sparidae	Demersal	0	0.00%	0	0.00%	2	0.02%	2	0.008%
Horsefish sp.		Congiopodidae	Demersal	0	0.00%	1	0.01%	1	0.01%	2	0.008%
Short alfonsino	*Centroberyx spinosus*	Berycidae	Bentho-pelagic	2	0.07%	0	0.00%	0	0.00%	2	0.008%
Brotulid sp.		Ophidiidae	Demersal	0	0.00%	1	0.01%	0	0.00%	1	0.004%
Greater pipefish	*Syngnathus acus*	Syngnathidae	Bentho-pelagic	0	0.00%	1	0.01%	0	0.00%	1	0.004%
Pilot fish	*Naucrates ductor*	Carangidae	Pelagic	0	0.00%	0	0.00%	1	0.01%	1	0.004%
Shadow driftfish	*Psenes whiteleggii*	Nomeidae	Bentho-pelagic	1	0.03%	0	0.00%	0	0.00%	1	0.004%
Slender snipefish	*Macroramphosus gracilis*	Centriscidae	Pelagic	1	0.03%	0	0.00%	0	0.00%	1	0.004%
Spotted greeneye	*Chloropthalamus punctatu*	Chlorophthalmidae	Demersal	0	0.00%	1	0.01%	0	0.00%	1	0.004%
Streepie	*Sarpa salpa*	Sparidae	Demersal	0	0.00%	1	0.01%	0	0.00%	1	0.004%
Snakehead toadfish	*Batrichthys apiatus*	Batrachoididae	Demersal	0	0.00%	0	0.00%	1	0.01%	1	0.004%
Trumpetfish sp.		Aulostomidae	Demersal	1	0.03%	0	0.00%	0	0.00%	1	0.004%
Unidentified larval fish				12	0.41%	10	0.10%	94	0.79%	116	0.469%
Unidentified fish				5	0.17%	6	0.06%	36	0.30%	47	0.190%
**Fish (Total)**				**2918**	98.78%	**9559**	98.16%	**11171**	93.76%	23648	96.127%
Cape Hope squid	*Loligo reynaudii*	Loliginidae	Pelagic	12	0.37%	31	0.32%	11	0.09%	54	0.214%
Cuttlefish sp.	*Sepia* sp.	Sepiidae	Pelagic	12	0.41%	36	0.37%	37	0.31%	85	0.344%
Octopus	*Octopus vulgaris*	Octopodidae	Bentho-pelagic	6	0.20%	1	0.01%	4	0.03%	11	0.044%
**Cephalopod (Total)**				**30**	1.02%	**77**	0.79%	**398**	3.34%	505	2.042%
Mantis shrimp	*Pterygosquilla armata capensis*	Squillidae	Demersal	3	0.10%	13	0.13%	228	1.91%	244	0.986%
Swimming crab		Brachyura **	Bentho-pelagic	0	0.00%	0	0.00%	3	0.03%	3	0.012%
Rock lobster	*Jasus lalandii*	Palinuridae	Demersal	0	0.00%	0	0.00%	3	0.03%	3	0.012%
**Crustaceans (Total)**				**3**	0.10%	**13**	0.13%	**234**	1.96%	250	1.007%
Two spotted cricket	*Gryllus bimaculatus*	Gryllidae	Terrestrial	3	0.10%	85	0.87%	103	0.86%	191	0.772%
African mole cricket	*Gryllotalpa africana*	Gryllotalpidae	Terrestrial	0	0.00%	1	0.01%	1	0.01%	2	0.008%
Butterfly/moth	Lepidoptera sp.	Sphingidae	Terrestrial	0	0.00%	0	0.00%	3	0.03%	3	0.012%
Beetle	Coleoptera sp.	Coleoptera *	Terrestrial	0	0.00%	0	0.00%	1	0.01%	1	0.004%
**Insect spp (Total)**		** **		**3**	0.10%	**89**	0.91%	**112**	0.94%	204	0.825%
**Totals**				2954		9738		11915		**24607**	

* Infraorder

** Order

#### Inter- and intra-annual variation of prey composition

The proportions of prey species in the diet of greater crested terns differed significantly between the three breeding seasons (χ^2^ = 2597.8, d.f. = 14, p < 0.001). In 2013, anchovy constituted 83% of the diet (by number), compared to 67% in 2014 and 62% in 2015, with redeye round-herring increasing from 3% in 2013 to 15% in 2015 (Table 1). The proportion of invertebrate prey in the diet also increased from 1% in 2013 to 6% in 2015. Although fish larvae contributed relatively little to the tern’s diet, they were most abundant in 2015 (0.8%, Table 2).

**Fig 1:**
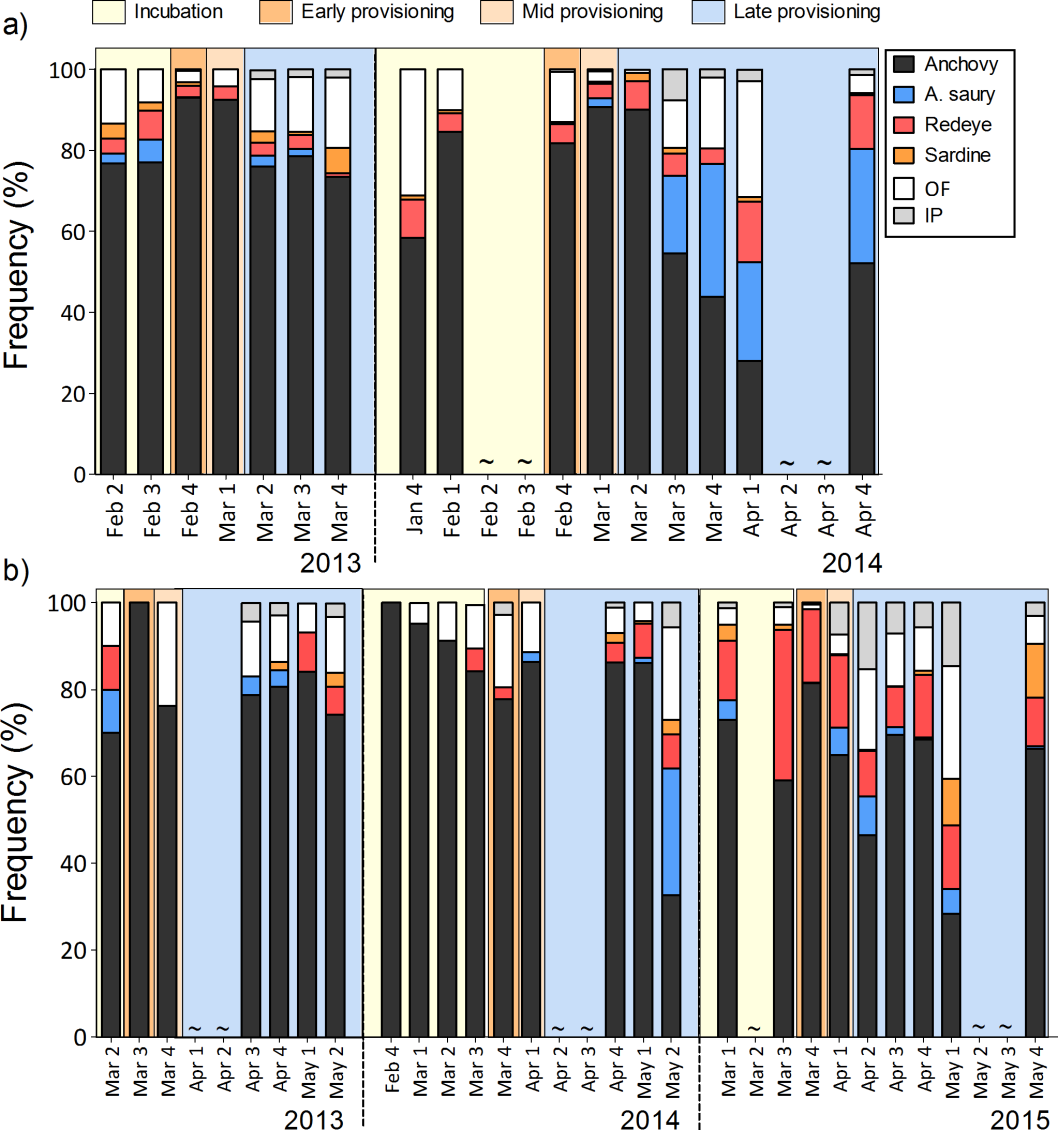
Fine-scale weekly variation in greater crested tern diet by mean frequency by number of the four major fish prey (anchovy, Atlantic saury, redeye round-herring and sardine), other fish (OF) and invertebrate prey (IP). Sampling locations were at the single-species colony (a) and mixed colony (b) at Robben Island across breeding seasons and stages. Breeding stages are illustrated as (i) incubation period, (ii) early, (iii) mid and (iv) late provisioning.

**Fig 2:**
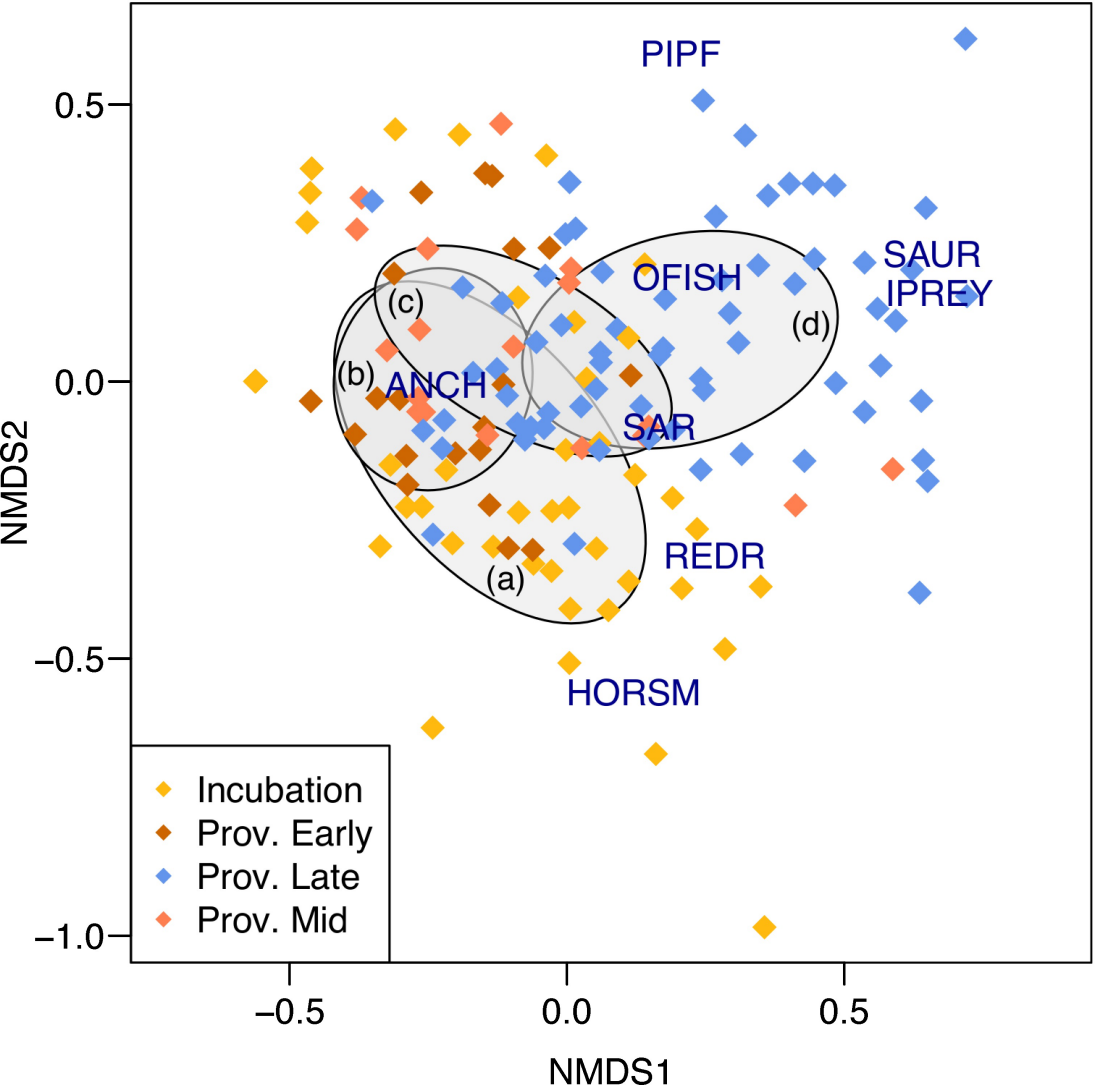
Non-metric multidimensional scaling plots (NMDS) of weekly mean proportions, showing differences in prey composition per breeding stage of greater crested tern diet across three breeding seasons (2013–2015) at both colonies at Robben Island. Sample points represent each sampled week divided by the stage of the colony (see legend). Position of points is related to the relative weekly contribution of each of the eight major prey groups. Grey shaded ellipses were used to highlight the centre of gravity of breeding phases: a) Incubation; b) Early provisioning; c) Mid provisioning; d) Late provisioning. (Prey groups: ANCH = Anchovy; REDR = Redeye round-herring; SAUR = Atlantic saury; HORSM = Horse mackerel; SAR = Sardine; PIPF = Pipefish; OFISH = other fish; IPREY = invertebrate prey).

**Fig 3:**
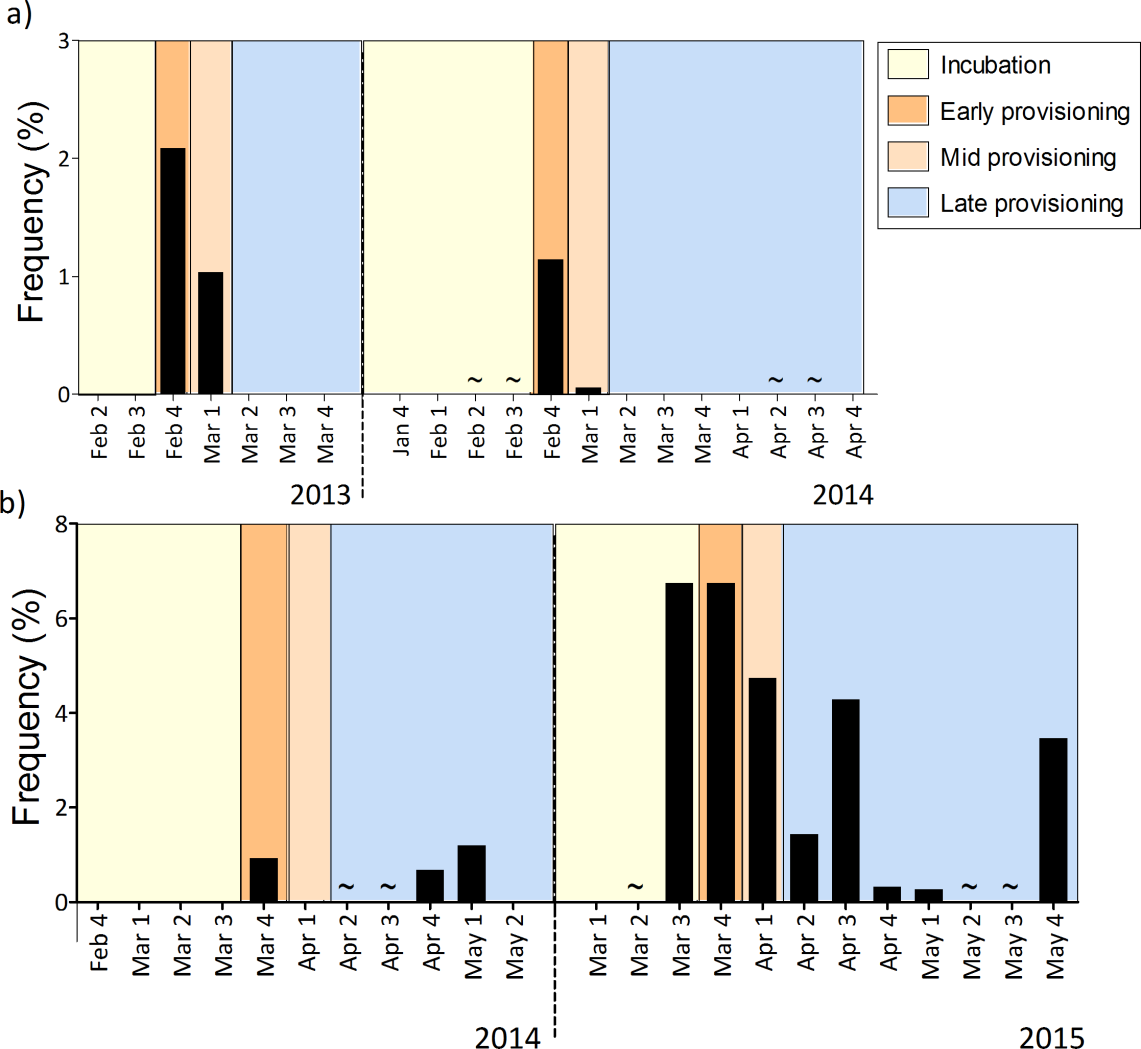
**Dietary contribution in terms of mean frequency of larvae** in (a) the single-species colony (2013–2014) and (b) the mixed colony (2014–2015) at Robben Island during different breeding stages, (i) incubation period, (ii) early, (iii) mid and (iv) late provisioning. * no data available from 2013 at mixed colony.

Anchovy were the most common prey during all breeding stages, but the diet was more diverse during incubation and late provisioning than when the adults were feeding small to mid-sized chicks (Figs 1 & 2). There was a significant difference in prey composition between prey returned for courtship or display during the incubation period and those returned during chick provisioning (χ^2^ = 998.55, d.f. = 6, p < 0.001, Figs 1 & 2). During incubation, in addition to anchovy, other pelagic species such as redeye round-herring (especially at the mixed colony during 2015) and horse mackerel were frequently brought back to the colony (Figs 1 & 2). Once the chicks hatched (early provisioning) the proportion of anchovy in the diet invariably increased, ranging between 80% and 100%, and there also was a greater proportion of fish larvae (Figs 1 & 3). During mid provisioning the diet was similar to that of the early provisioning stage (Figs 1 & 2), but the proportion of fish larvae decreased (Fig 3). During late provisioning, the diet became more diverse, including larger fish species, such as Atlantic saury, redeye round-herring and sardine, as well as invertebrate prey (Figs 1 & 2).

### Prey composition in relation to environmental factors

**Fig 4:**
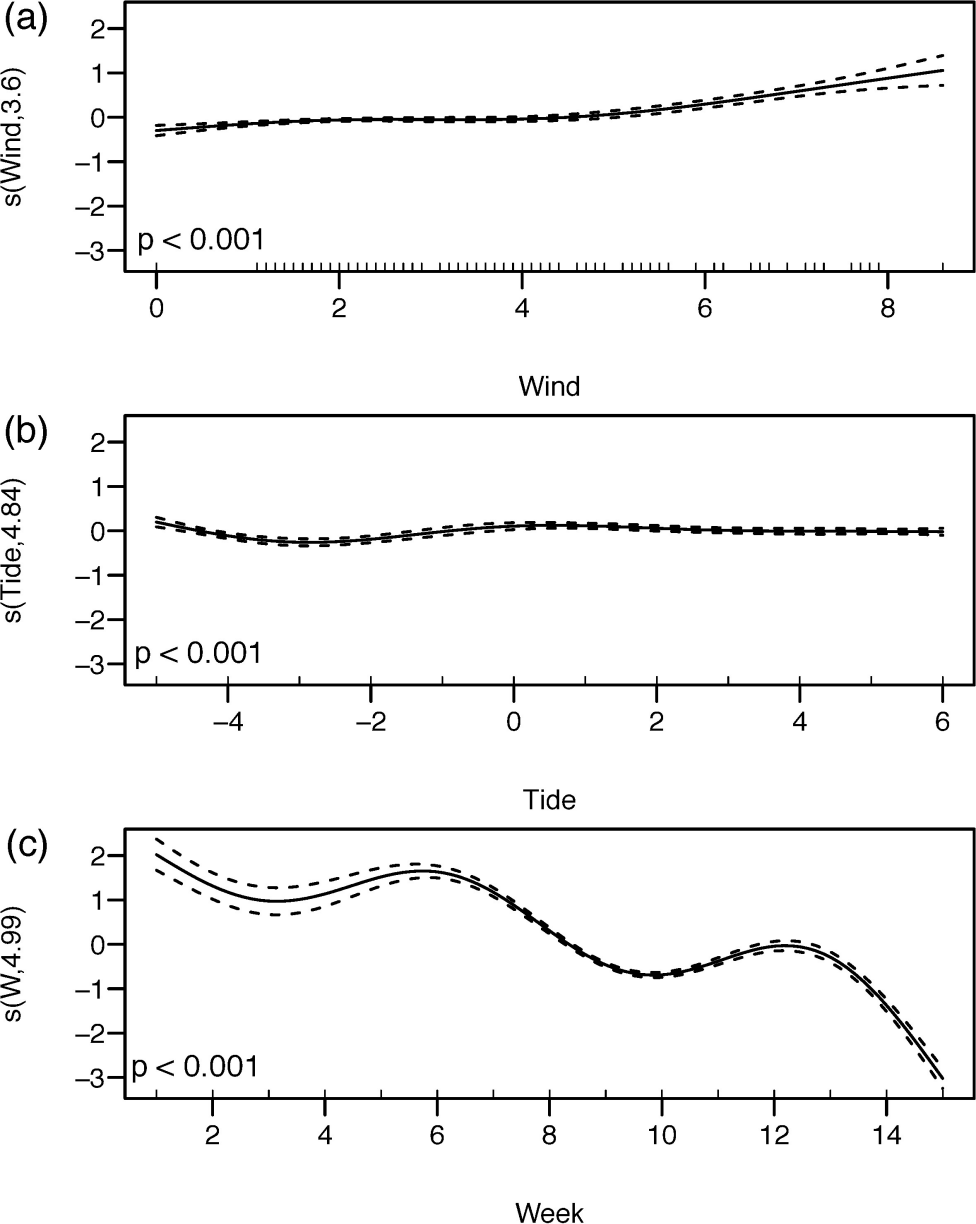
**GAM partial predictions for the probability of anchovy occurring in the greater crested terns’ diet according to (a) wind velocity, (b) tide and (c) week from the onset of breeding.** Tidal stage (b) is defined as hours before and after high tide (high tide = 0). Week (c) represents the sequence of weeks commencing from the last week of January (1) until the 2^nd^ week of May (15). Data are from the 2013–2015 breeding seasons on Robben Island. s(x,y) is the smoothing term, with x the explanatory variable and y the estimated degrees of freedom of the smoothing term. The pointwise 95% confidence intervals are shown as dashed lines for each model fit.

Tests of covariance between all continuous variables used in the GAMs all had variance inflation factors < 3, so all variables were included in the model. There was a positive relationship between wind velocities > 5 m s^-1^ on the proportion of anchovy returned to the colony (edf = 3.6, χ^2^ = 123.9 p < 0.001, Fig 4). The influence of tide, although significant, was very weak, with slightly higher probabilities of anchovy returned during the first peak of low tide (-5 hrs) and in between low and high tides (3 hrs) (edf = 4.8, χ^2^ = 53.23 p < 0.001, Fig 4). The proportion of anchovy returned to the colony generally decreased over the course of the breeding season, with a first peak of anchovy occurring during week 6 (1^st^ week of March) and a second peak occurring during week 12 (3^rd^ week of April) (edf = 4.9, χ^2^ = 1309.5 p < 0.001, Fig 4). Visibility had a negative influence on the proportion of anchovy returned with a 59% lower probability of returning anchovy during foggy weather (Table 3). More anchovies were returned to the colony in 2013 than 2014 or 2015, and when both colonies were active during 2013 and 2014, anchovy were returned to the single-species colony with a lower probability than to the mixed-species colony (Table 3).

**Table 3:** Parametric coefficients of fixed effects used in the generalised additive models (GAM) used to assess the influence of non-linear variables (wind velocity, tide and week) on the presence of anchovy in the diet of greater crested terns.

Model term	Estimates	Std. Error	P value
Intercept	3.01	0.096	**< 0.001**
Weather (foggy)	-0.90	0.066	**< 0.001**
Year (2014)	-0.70	0.063	**< 0.001**
Year (2015)	-1.71	0.081	**< 0.001**
Colony (single-species)	-1.61	0.085	**< 0.001**
Breeding stage (incubation)	-1.44	0.102	**< 0.001**
Breeding stage (late)	-0.39	0.100	**< 0.001**
Breeding stage (mid)	-0.06	0.079	0.41

### Anchovy size

**Fig 5:**
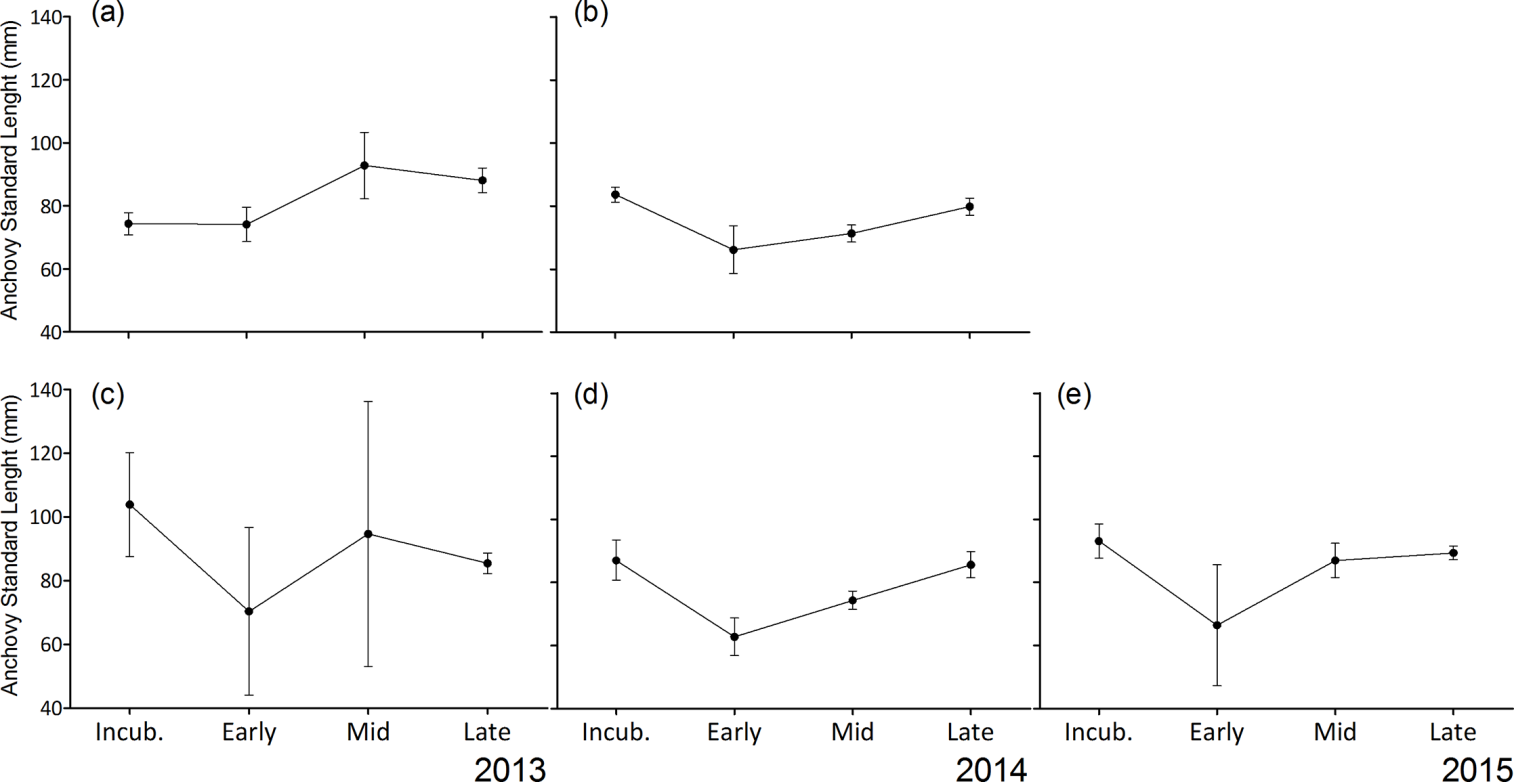
Anchovy standard length in the diet of greater crested terns across four breeding stages. Breeding phases are the incubation period (Incub.), early- (Early), mid- (Mid) and late-provisioning (Late) periods. Data are from the single-species colony (a) in 2013 and (b) in 2014, at the mixed colony (c) in 2013 and (d) in 2014, and (e) in 2015 across breeding stages in Robben Island.

Excluding larvae, anchovy standard length ranged from 32 mm to 125 mm (mean ± SD for all years combined: 83.9 ± 16.8 mm). Inter-annual differences in anchovy SL were significant (ANOVA: F_(2,883)_ = 81.48, p < 0.001, n = 886) with larger anchovies caught in 2015 (mean ± SD: 92 ± 16 mm) than in 2013 (84 ± 16 mm) or 2014 (76 ± 15 mm) (Tukey test, p < 0.001). The anchovy brought back during incubation at both colonies were significantly larger (mean ± SD: 85 ± 13 mm) (Table 4) than those recorded during early and mid provisioning (mean ± SD, early: 77 ± 20 mm; mid: 74 ± 15 mm), but similar in size to those delivered during late provisioning (mean ± SD, 85 ± 14 mm; Table 4). Results were similar when considering the two colonies separately, but differences were only significant at the mixed-species colony (Table 4, Fig 5).

**Table 4:** Mean (SD and Tukey test adjusted p-values from comparisons of anchovy standard length (mm) estimated from photo-samples compared across breeding stages and colonies in all years at Robben Island. Breeding phases are referred as (i) incubation period, (ii) early, (iii) mid and (iv) late provisioning.

	Both colonies combined				Single-species colony				Mixed-species colony			
		P adj				P adj				P adj		
Breeding stage	mean (SD) mm	Early	Mid	Late	mean (SD) mm	Early	Mid	Late	mean (SD) mm	Early	Mid	Late
**Incubation**	85 (13)	**< 0.01**	**< 0.01**	0.99	80 (9)	0.32	0.02	0.99	92 (14)	**< 0.01**	**< 0.01**	0.78
**Early**	77 (20)	-	0.93	**< 0.01**	74 (12)	-	0.99	0.14	77 (21)	-	0.99	**< 0.01**
**Mid**	74 (15)	-	-	**< 0.01**	74 (13)	-	-	**< 0.01**	76 (15)	-	-	**< 0.01**
**Late**	85 (14)	-	-	-	t82 (14)	-	-	-	88 (14)	-	-	-

## Discussion

Our study describes the prey exploited by the largest breeding aggregation of greater crested terns in southern Africa during three successive breeding seasons. As for previous studies in the Western Cape, greater crested terns mainly targeted small schooling Clupeiformes, primarily relying on anchovy and, to a lesser extent, redeye round-herring, two of the most common mid-trophic fish species in the Benguela upwelling region [23, 44]. Despite the dominance of Clupeiformes in their diet during this study, greater crested terns demonstrated considerable foraging plasticity with the ability to capture demersal fish as well as cephalopods and arthropods (Table 1 and Table 2, [45]). Previous knowledge of greater crested tern diet in southern Africa came from the collection of chick regurgitations and observations of prey fed to chicks [20, 21]. However, both these methods are subject to methodological limitations, including biases introduced by differential prey regurgitation [46] or the potential misidentification of prey based solely on instantaneous observations [47]. Our study gives novel information on prey composition, with insights into flexible foraging strategies.

### Prey diversity and prey ecology

Our photo-sampling technique revealed at least 28 new species of fish, one cephalopod, two crustaceans and three species of insects previously not recorded in the tern’s diet. A large diversity of prey also has been reported in the diets of greater crested terns in Australia [19, 48], reflecting this species’ considerable foraging plasticity. Breeding greater crested terns rely mostly on anchovy, which is an abundant pelagic fish in the southern Benguela system and provides high energetic value for growing chicks [22, 49, 50]. After anchovy, the second most abundant prey in the tern’s diet was redeye round-herring, which is also an important mid-trophic level species in this system [22, 51]. It was particularly abundant in the diet during mid to late chick provisioning. During the day, redeye schools descend into deeper water [12], but their presence in the tern’s diet may be explained by the fact that juvenile fish are found near the surface in inshore waters where they form mixed schools with anchovy and juvenile sardine [51]. In addition, other predators such as African penguins or dolphins, may force redeyes and other mid-water species, such as lantern fish *Lampanyctodes hectorus*, to the surface, making them available to terns [52, 53]. Atlantic saury were particularly abundant in the diet when adults were provisioning fledglings, as observed in Cape gannets [5] and roseate terns *Sterna dougalli* in the Azores [54]. Due to their relatively large size and high energy content [49], saury are ideal prey to be fed to large chicks. Although saury normally have an offshore distribution, longitudinal migration of this species, which brings shoals closer inshore during autumn [55], may increase availability to terns during the later stages of the breeding season.

Long-snout pipefish, a widespread species in coastal waters [56], were recorded for the first time in the diet of greater crested terns. Although often associated with benthic habitats, it can occur in mid water. Other species, such as redfingers *Cheilodactylus fasciatus*, rocksuckers *Chorisochismus dentex*, blennidae, clinidae or soleidae, which are bottom dwellers, are likely captured in shallow coastal habitats, including tidal pools. Some of the demersal fish species recorded live near the sea surface as juveniles, including beaked sandfish and bluebottle fish *Nomeus gronovii* [56], and are captured by terns at this life stage. Like beaked sandfish, juvenile Cape hake *Merluccius capensis* can occur near the surface during the daytime [57, 58]. Small hake may be captured early in the morning as they migrate to the surface at night to feed on fish and crustaceans [56]. The occasional presence of mantis shrimps *Pterygosquilla armata* in the tern’s diet likely results from these crustaceans periodically migrating into surface waters [59] and the occasional presence of terrestrial prey (e.g., crickets) suggests that insects may be captured opportunistically when they aggregate in the vicinity of the colony [45].

### Factors affecting the temporal variation in greater crested tern diet

A key strength of our study comes from monitoring tern diet throughout the breeding season, which provided an index of the relative abundance of prey types and sizes of anchovy. However, this index incorporates some amount of selection by terns on prey type and size returned to the colony. The proportions and sizes of the main prey varied between incubation and chick provisioning. Small anchovies were most abundant during the first phases of provisioning (early: SL = 77 ± 20 mm; mid: SL = 74 ± 15 mm), compared to late provisioning when other large species were also abundant and anchovy SL was significantly larger (85 ± 14 mm). These results support the ontogenetic differences in food requirements between adults and chicks reported for greater crested terns found in Australia [9] and for other seabird species (e.g., [47, 60–62]). The preponderance of small anchovies and fish larvae delivered during early chick provisioning presumably are related to the limited gape and gut capacity of hatchlings, but still provide chicks with the benefits of high calorific value prey [49]. In Australia, the Australian anchovy *Engraulis australis* and sardine were the most abundant prey of breeding greater crested terns and a comparison with chick regurgitations confirmed that prey were smaller on average during early provisioning than when chicks were larger and more mobile [19]. Delivering larger prey to large chicks reflects the increased energetic demands at this stage, when parents have to maximise the efficiency of each foraging trip, bringing back as much food as possible as predicted by central place foraging models for single prey loaders [10, 63, 64].

Our study suggests that adult prey choice is influenced by their chick’s stage, but prey choice also is influenced by the spatio-temporal availability of prey (e.g., [65]) and the extent to which this may influence the greater crested terns’ diet should not be discounted. Most anchovy delivered by greater crested terns were recruits (SL < 100 mm), which were recorded during all breeding stages. Based on the movements of anchovy recruits in the Benguela region, young-of-the-year anchovy would only be expected to reach Robben Island after May [40, 41, 66], i.e., toward the end of the greater crested tern breeding season when most chicks have fledged. Our findings contradict this general trend, with anchovy (mostly recruits) decreasing in abundance as the breeding season progressed from February to May. During late provisioning, parents could have buffered scarcity of anchovy by targeting alternative available prey, some of which are known to move closer to shore at that time (e.g., Atlantic saury, [55]) or which could be found at more distant feeding grounds, as parents generally perform longer foraging trips during this period [67]. The foraging range of greater crested terns breeding in southern Africa is currently unknown; but is assumed to be ca 10 km [20]. This restricted spatial range in which terns acquire their food, combined with competition with other short-range foragers which breed concurrently at Robben Island (e.g., African penguin; foraging range ca 40 km) may result in a local depletion of resources, referred to as ‘Ashmole’s halo’ [68]. However, the magnitude of the terns’ impacts on anchovy stocks around the breeding colony are unknown.

Breeding is often timed to coincide with the peak availability of food [69], either during the weeks leading up to breeding, when adult body condition can play a significant role in mediating breeding success [70, 71], or during subsequent breeding stages when food supplies are critical for chick or fledgling survival [72]. Unfortunately, there are scant data on the temporal abundance of anchovy around Robben Island during the terns’ breeding season [73]. Based on monthly counts of anchovy and sardine eggs and larvae ca 30 km south of Robben Island between 1995 and 2001, [40] reported a clear peak in the abundance of anchovy larvae during January and February, which may influence tern breeding phenology.

Prey choice by visual predators may be influenced by physical conditions while foraging [74]. For example, strong winds increase prey catch rates in sandwich *Thalasseus sandvicensis*, common *Sterna hirundo* and Damara terns *Sternula balaenarum* [75, 76]. Our study found that the proportion of anchovy increased at wind speeds > 5 m s^-1^. Strong winds may elevate zooplankton in the water column and influence the vertical location of anchovy [77, 78], or increase flight efficiency allowing terns to travel further where more anchovy may be available. Alternatively, at high wind speeds there is reduced detection of prey, hence terns have to rely on other cues for prey location (e.g., through local enhancement, [79, 80]). In the Western Cape, greater crested terns are likely to frequently encounter Cape cormorants and African penguins foraging on anchovy [21]. Therefore, the positive correlation between the proportion of anchovy in the diet and increased wind speeds may be linked to the prey preferences of these other predators. The smaller proportion of anchovy returned to the colony on foggy days (Table 3, Fig 4) suggests that terns struggle to find anchovy schools when visibility is poor because they are less able to detect heterospecific feeding groups, necessitating a switch to a more opportunistic foraging behaviour [81].

### Coping in an exploited environment

Despite anchovy dominating the diet of greater crested terns during the breeding season, our results demonstrate the terns’ ability to take advantage of feeding opportunities on prey that is less abundant or has a lower energetic value [45, 82] Such foraging plasticity in combination with their varied foraging methods (e.g., plunge diving, surface-seizing, scavenging, kletpoparasitism, diving from perches, taking prey in flight as well as from off the ground) [17, 45, 82], may be a key characteristic in locations where Clupeiformes have become scarce and patchily distributed at small temporal and spatial scales [83]. For example, in the northern Benguela off Namibia, terns predominantly feed their chicks juvenile bearded goby *Sufflogobius bibarbatus* (J.-P. Roux pers. comm.), which is locally abundant, albeit low in energetic content [4]. This adaptability may underpin the tern’s capacity to cope in a highly dynamic and heterogeneous environment such as the Benguela upwelling region [84]. However, as the Benguela ecosystem has been recently exposed to considerable modification resulting from environmental changes and anthropogenic effects [22] it is difficult to disentangle the influence of synergistic changes (e.g., climate change and fisheries; [85]). The foraging plasticity of the greater crested tern may be postulated as a short-term response to climate change; a key factor that allows this predator to thrive in comparison to other more specialist seabirds.

## Conclusions

This is the first investigation of diet in a single prey-loading seabird using a method that allows for intensive sampling over protracted periods with little disturbance [31]. The study greatly increased our knowledge of prey caught by the southern African population of greater crested terns during the breeding season, offering new insights into the relationships between predators and prey in the Benguela upwelling region. Flexible foraging strategies are often the most effective short-term responses to climate change and may buffer predators against the uncertainty of locating food in a stochastic and exploited system such as the Benguela region [28]. In such areas, due to their restricted foraging range when breeding and the availability of a real-time monitoring method with limited sampling effort and disturbance [86], greater crested terns may be important ocean sentinels indicating localized human-induced alterations in common and non-commercialized species. The non-invasive method used in this study offers a fine-scale window into their diet and behaviour that can be implemented to undertake systematic and detailed monitoring of changes in the availability of prey in the vicinity of breeding colonies. Comprehensive long-term diet studies will contribute to our understanding of how greater crested terns cope with the apparent variation in food availability better than other seabird species relying on the same primary food resource and breeding in the same marine ecosystem.

## Supporting information

S1 Table Time spent (in hours) photo-sampling for each week of the month. Sampling data is shown per colony and years.
(PDF)

S1 Fig Example of camera-trap photographs recording the breeding stage of a subsample of nests within the colony. (*Top*) Photograph recorded on the 03/23/2015 at 9:27:04 showing an adult sitting on an egg. (*Bottom*) photograph of the same nest recorded on the same day 3.5 minutes later, showing the egg has hatched.
(TIF)

S2 Fig Examples of prey returned to the colony by adult greater crested terns. From A to N: A) klipfish *Clinid* sp.; B) octopus *Octopus vulgaris*; C) Cape hope squid *Loligo vulgaris reynaudii*; D) rocksucker *Chorisochismus dentex*; E) spotted greeneye *Chloropthalamus punctatus*; F) greater pipefish *Syngnathus acus*; G) kingklip *Genypterus capensis*; H) redfingers *Cheilodactylus fasciatus*; I) southern conger *Gnathophis capensis*; J) crab Brachyura; K) toadfish *Batrichthy sapiatus*; L) hawk-moth Sphingidae; M) grenadier Macrouridae; N) silver scabbardfish *Lepidopus caudatus*.
(TIF)
