# Supplementary material for: Foraging plasticity in seabirds: A non-invasive study of the diet of greater crested terns breeding in the Benguela region
Source: PLoS One. 2018 Jan 31;13(1):e0190444. doi: 10.1371/journal.pone.0190444 (PMC5791960; doi:10.1371/journal.pone.0190444)
Supplement: S1 Table — Sampling data is shown per colony and years. (PDF) [file pone.0190444.s001.pdf]

S1 Table: Time spent (in hours) photo-sampling for each week of the month. Sampling data is shown per colony and years.

Colony		Single-species	Mixed
Year	Week of the month	Hours of sampling	
2013	February 2	2.5	-
	February 3	12.0	-
	February 4	16.2	-
	March 1	1.6	-
	March 2	2.1	2.4
	March 3	9.0	2.4
	March 4	4.5	2.0
	April 3	-	2.0
	April 4	-	3.1
	May 1	-	2.0
	May 2	-	2.0
2014	January 4	3.0	-
	February 1	4.1	-
	February 4	4.0	1.1
	March 1	6.7	2.1
	March 2	4.1	1.0
	March 3	5.0	1.2
	March 4	3.2	6.0
	April 1	5.4	4.4
	April 4	4.2	4.0
	May 1	-	5.0
	May 2	-	1.3
2015	March 1	5.6	NA
	March 3	10.1	NA
	March 4	5.3	NA
	April 1	13.1	NA
	April 2	3.0	NA
	April 3	7.1	NA
	April 4	11.8	NA
	May 1	7.5	NA
	May 2	12.5	NA
