# Supplementary figures and images for: Foraging plasticity in seabirds: A non-invasive study of the diet of greater crested terns breeding in the Benguela region
Source: PLoS One. 2018 Jan 31;13(1):e0190444. doi: 10.1371/journal.pone.0190444 (PMC5791960; doi:10.1371/journal.pone.0190444)

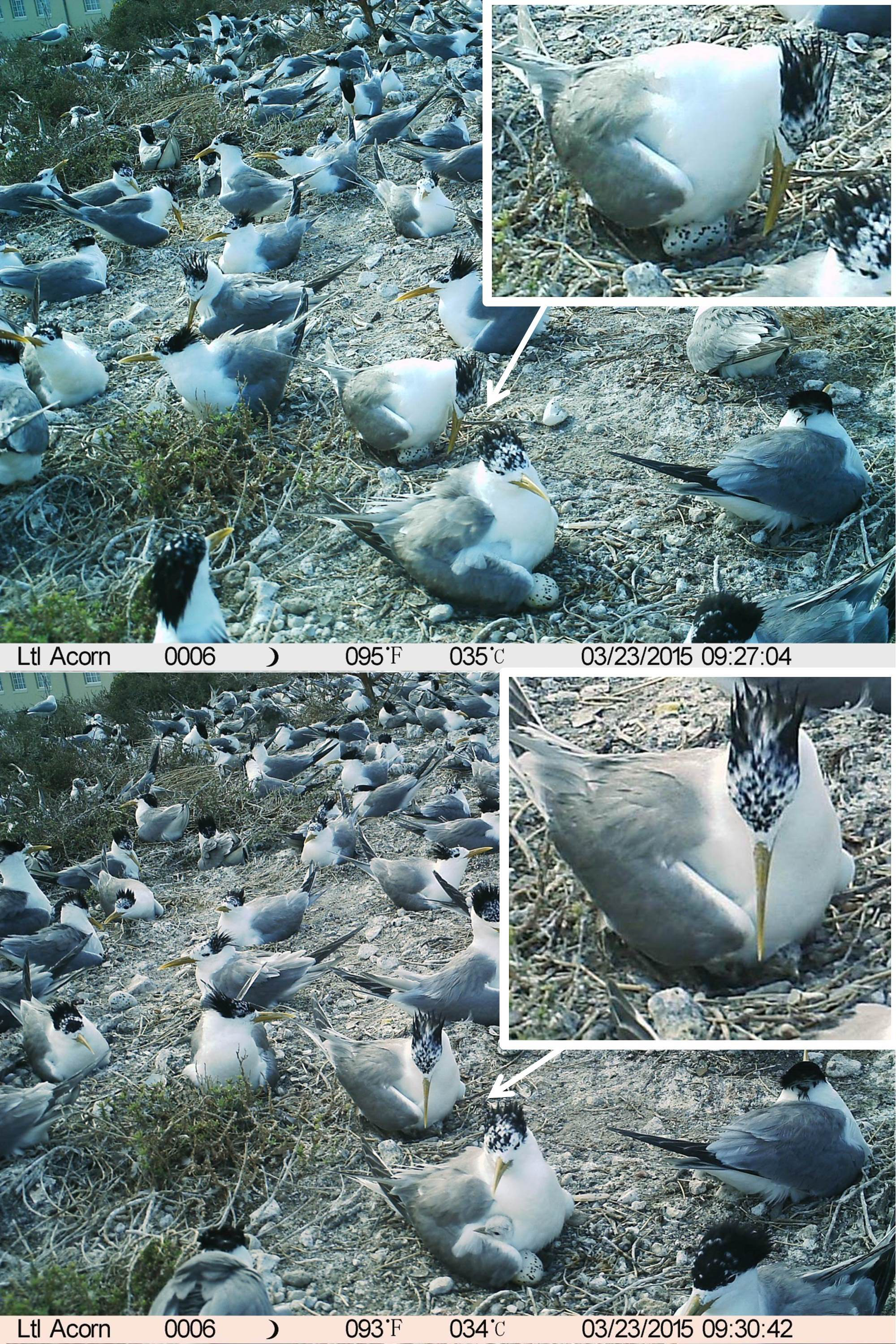

Supplement: S1 Fig — (Top) Photograph recorded on the 03/23/2015 at 9:27:04 showing an adult sitting on an egg. (Bottom) photograph of the same nest recorded on the same day 3.5 minutes later, showing the egg has hatched. (TIF) [file pone.0190444.s002.tif]

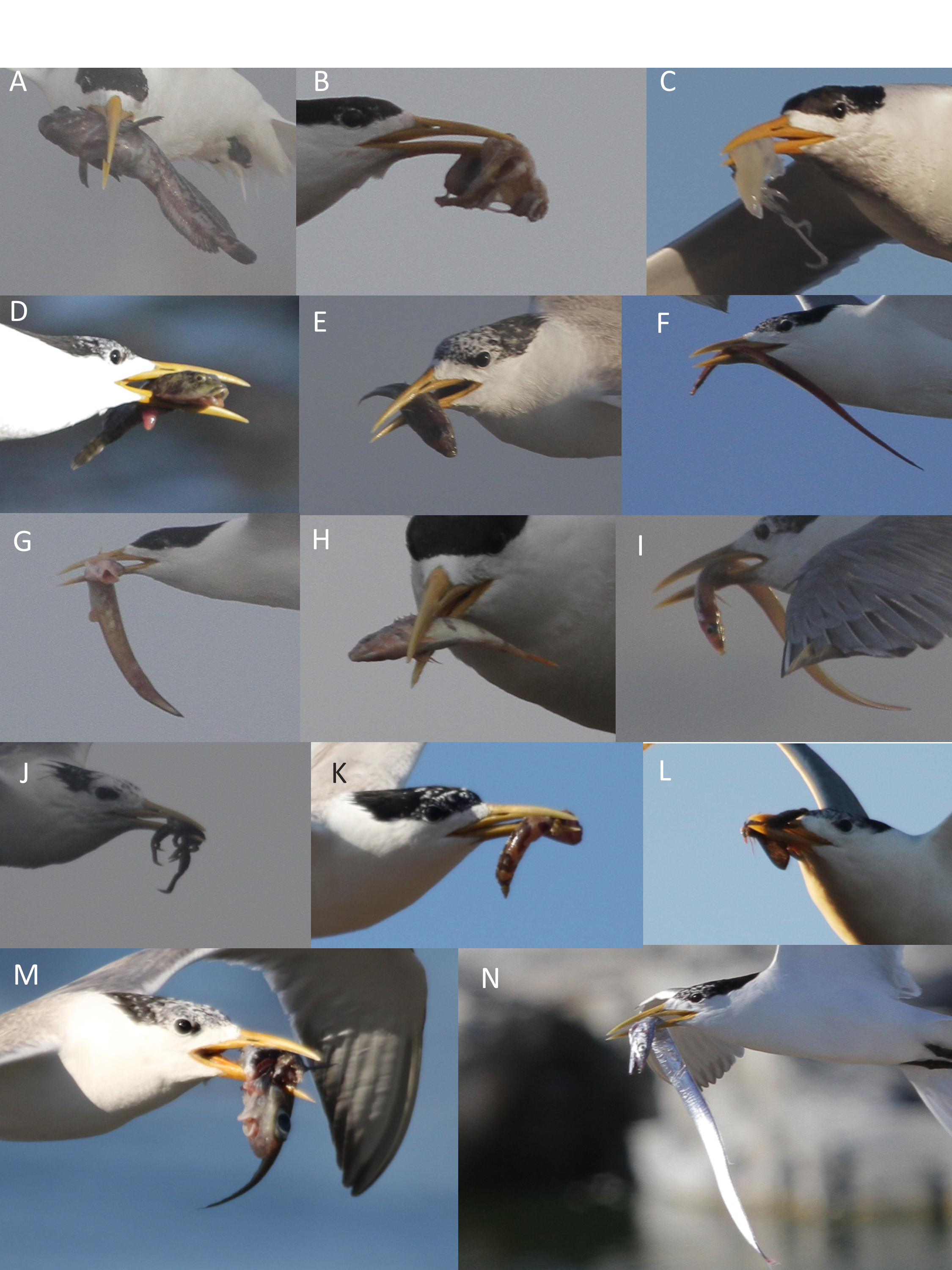

Supplement: S2 Fig — From A to N: A) klipfish Clinid sp.; B) octopus Octopus vulgaris; C) Cape hope squid Loligo vulgaris reynaudii; D) rocksucker Chorisochismus dentex; E) spotted greeneye Chloropthalamus punctatus; F) greater pipefish Syngnathus acus; G) kingklip Genypterus capensis; H) redfingers Cheilodactylus fasciatus; I) southern conger Gnathophis capensis; J) crab Brachyura; K) toadfish Batrichthy sapiatus; L) hawk-moth Sphingidae; M) grenadier Macrouridae; N) silver scabbardfish Lepidopus caudatus. (TIF) [file pone.0190444.s003.tif]
